# Functional connectivity patterns in parosmia

**DOI:** 10.1186/s12993-023-00225-8

**Published:** 2023-12-19

**Authors:** Divesh Thaploo, Akshita Joshi, Eren Yilmaz, Duzgun Yildirim, Aytug Altundag, Thomas Hummel

**Affiliations:** 1 Smell & Taste Clinic, Department of Otorhinolaryngology, Universitätsklinikum Carl Gustav Carus, Technische Universität Dresden, Fetscherstrasse 74, 01307 Dresden, Germany; 2 Faculty of Health Sciences, Istanbul Gelisim University, Istanbul, Turkey; 3 Department of Medical Imaging, Acibadem University, Vocational School of Health Sciences, Istanbul, Turkey; 4 Faculty of Medicine, Department of Otorhinolaryngology, Biruni University, Istanbul, Turkey

**Keywords:** Parosmia, Functional connectivity, Hyposmia, Salience, Executive control

## Abstract

**Objective:**

Parosmia is a qualitative olfactory dysfunction presenting as “distorted odor perception” in presence of an odor source. Aim of this study was to use resting state functional connectivity to gain more information on the alteration of olfactory processing at the level of the central nervous system level.

**Methods:**

A cross sectional study was performed in 145 patients with parosmia (age range 20–76 years; 90 women). Presence and degree of parosmia was diagnosed on the basis of standardized questionnaires. Participants also received olfactory testing using the “Sniffin’ Sticks”. Then they underwent resting state scans using a 3 T magnetic resonance imaging scanner while fixating on a cross.

**Results:**

Whole brain analyses revealed reduced functional connectivity in salience as well as executive control networks. Region of interest-based analyses also supported reduced functional connectivity measures between primary and secondary olfactory eloquent areas (temporal pole, supramarginal gyrus and right orbitofrontal cortex; dorso-lateral pre-frontal cortex and the right piriform cortex).

**Conclusions:**

Participants with parosmia exhibited a reduced information flow between memory, decision making centers, and primary and secondary olfactory areas.

## Introduction

Causes of OD are diverse and range from infections of the upper respiratory tract, traumatic brain injury, and chronic rhinosinusitis to neurodegenerative diseases, like Parkinson’s disease [1, 2]. OD can be broadly divided into two types of impairments, quantitative (hyposmia or anosmia) and qualitative (parosmia or phantosmia). Parosmia is defined as a distorted sense of smell in presence of an odor source whereas the latter refers to odorous impressions in the absence of an odor source [3]. What sets parosmia apart from quantitative olfactory dysfunctions is that individuals with parosmia are more vividly reminded of these distorted or unpleasant smells. In contrast, many patients with quantitative olfactory dysfunction can adjust to the loss with time [4, 5].

Since it is not possible to objectively measure qualitative disorders, physicians have to rely on the interview with the patients supported by structured questionnaires. In this direction, a 4-item questionnaire is widely used to determine a so-called parosmia score [6]. The questions focus on intensity, frequency and the effect of OD on flavor perception. Other ways to grade the severity of parosmia utilize questions on intensity of parosmia, frequency of parosmic sensations, and significant effects on weight, life style, or eating habits. A score estimating the degree of parosmia is calculated as the sum of parosmia intensity (not very intense = 0, very intense = 1), parosmia frequency (not daily = 0, daily = 1), and significant parosmia consequences (not present = 0, present = 1) [7].

**Fig. 1:**
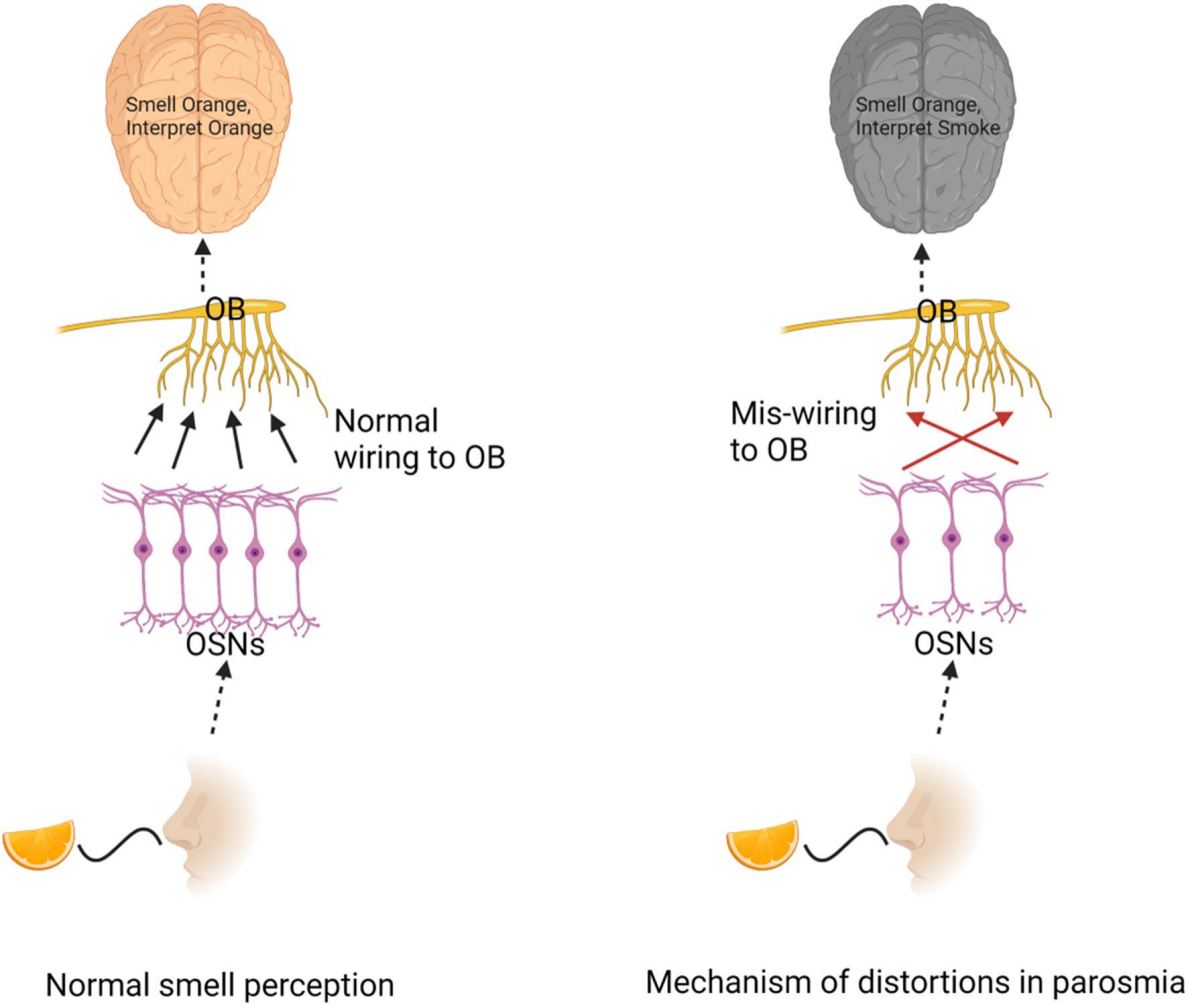
Proposed mechanism for parosmia. Comparison between normal smell processing pathway and distorted perception in parosmia. Smelling an orange would trigger a cascade of reactions via OSNs and travelling all the way to OBs which in turn would project orange smell in the brain. However, in parosmia, due to loss of OSNs and mis-wiring, distorted perception of orange as smoke in the brain. OB, olfactory bulb, OSNs, olfactory sensory neurons

The pathophysiology of parosmia is unclear. It has been attributed to a distorted coding of odor qualities based on the idea that some but not all olfactory neurons are present in order to form an incomplete pattern which would incorrectly encode the odor [8]. Although such distortions are usually unpleasant and disgusting, cases have been reported where patients describe the odor distortions as pleasant [9]. In addition to the “peripheral theory” of parosmia [10] it has been suggested that parosmia could also result from incomplete coding of odors on a central nervous level where integration of information is incomplete which is referred to as the “central theory” (Fig. 1) [2]. The peripheral theory in principle states that due to miswiring at the level of olfactory sensory neurons, a distorted perception result. On the other hand, the central theory states that distortions could result from a lack of coordinated information sharing between various higher order regions and olfactory areas [11]. However, there is no strong evidence of either and this makes it more interesting to have a longitudinal study design to examine this phenomenon further.

Patients suffering from parosmia report deteriorated quality of life as day-to-day activity like showering or oral care can become a challenge. Some patients also report being anxious about their future [12] while others change their diets leading to significant changes in their weight [13]. Such changes have been frequently reported with COVID-19 where parosmia often occurs during the recovery phase when patients start regaining their sense of smell [12, 14].

Functional magnetic resonance imaging (fMRI) is a non-invasive technique to study brain functions in the presence or absence of a task [15]. Resting state fMRI, also known as task free, is a technique to measure blood oxygenated level dependent (BOLD) signals spontaneously and is particularly attractive for patient populations as no task related activities have to be performed which might be complicated for some patients with neurological, neurosurgical, psychiatric conditions and older people in general [16]. Whole brain FC using independent component analysis (ICA) is an effective way to analyze low frequency fluctuations (< 0.1 Hz) from resting state fMRI. ICA helps in effective extraction of distinct areas in the brain (networks), by decomposing them into distinct temporal and spatial components [17]. There is some growing criticism about the use of denoising techniques in resting state fMRI, especially related to AROMA. However, AROMA still is one of the reliable measures to denoise fMRI data and when comparing with other algorithms like ICA-FIX or aCompCor, AROMA seems to perform at par [18, 19]. It must also be noted that as per the developers, AROMA must be implemented after performing pre-processing with FEAT FMRIB toolkit [20] for better ICA classification. There are very few studies about resting state fMRI in olfaction as compared to other senses and there are even less in patients with olfactory dysfunction. Still, results from the existing literature found altered inter-network functional connectivity (FC) in olfactory networks in patients suffering from post-traumatic anosmia [21]. One recent study in a COVID-19 affected population [22] found increased FC between orbitofrontal cortex (OFC) and both anterior and posterior piriform cortex. However, the sample size was low (N = 40) thus results of this elegant study have to be interpreted with care.

Using resting state fMRI, aim of the present study was to examine brain connectivity in patients suffering from parosmia. To understand FC patterns in different degrees of parosmia, we used two approaches. Firstly, whole-brain FC patterns, carried out using group ICA, were used to compare groups where we expected decreased FC among higher degree of parosmia. Secondly, we used a region-based approach, using OFC and piriform cortex as seeds and then compared the FC patterns among groups (compare [22]). These regions are primarily referred as the secondary and primary olfactory areas and we expected alterations in these regions in different degrees of parosmia. To define different degrees of parosmia, we used available questionnaires [6, 7].

## Methods

*Patient recruitment*A cross-sectional study involving 145 patients experiencing post-COVID olfactory dysfunction (OD) with varying degrees of parosmia (grades 0–3) was conducted. All participants were duly informed about the study, and written consent was obtained from each of them. Recruitment of these participants took place during the COVID-19 pandemic, a period marked by limited available resources. Post-hoc sample size estimations were performed considering a medium effect size of 0.5, α (significance level) set at 0.05, and a statistical power of 0.95.

This study received approval from the Institutional Review Board of Acibadem Mehmet Ali Aydinlar University, Istanbul, under the application number 2022–12/16.

*Olfactory testing* All patients received an otorhinolaryngological examination including nasal endoscopy, a thorough history and odor threshold testing using the standardized “Sniffin’ Sticks” based on pen-like odor dispensers [23]. For the olfactory threshold test, the rose-like odor phenylethyl alcohol was used. The test was executed in the staircase-procedure and consisted of 16 triplets of odor pens. One pen of each triplet contained phenylethyl alcohol at a certain dilution while the other two pens were odorless [23]. Because threshold testing is cognitively less demanding and, in comparison to odor identification, provides more information on the peripheral olfactory function, we did not perform the complete “Sniffin’ Sticks” battery [24]. A recent publication emphasized the importance of threshold test to better infer olfactory function [25].

### Definition of groups

Different degrees of parosmia were defined based on the responses to the 3 questions described above [7]. Parosmia degree 0 (Par 0) is defined as the group suffering from olfactory loss and no parosmia like symptoms, essentially the control group. Degree 1 (Par 1) refers to patients experiencing any one of the symptoms as described in the questionnaire. Par 1 patients developed parosmia 37 ± 19 weeks post anosmia or hyposmia. Degree 2 (Par 2) refers to patients exhibiting 2 symptoms whereas degree 3 (Par 3) includes patients with all the 3 symptoms. Since the number of patients suffering from Par 3 was small, we joined the Par 3 group and the Par 2 group, and labelled it as Par 2–3. Par 2–3 patients developed parosmia 40 ± 21 weeks post anosmia or hyposmia (Table 1). We did not control for duration of hyposmia as olfactory sensitivity was not significantly different between the parosmia groups as seen using ANOVA *(F (2, 141)* = *1.23, p* = *0.45)*.

**Table 1:** Demographic table

Groups	No of participants	Age (Mean ± SD)	Threshold scores (Mean ± SD)	Hyposmia duration in months (Mean ± SD)	Parosmia duration in weeks after anosmia/hyposmia (Mean ± SD)
Par 0	49, 30 females	41.2 ± 16.2	4.4 ± 1.8	9.7 ± 6	N.A
Par 1	45, 25 females	42.7 ± 14.6	4.3 ± 2	10.5 ± 4.3	9 ± 1.5
Par 2–3	51, 35 females	42.5 ± 13.5	4.0 ± 1.9	11.2 ± 5	8.8 ± 1.4

*SD*, standard deviation

### Imaging paradigms

All scans were carried out using a 3 T MRI scanner (type Numaris; Siemens, Erlangen, Germany). A resting state functional scan using single shot echo-planar imaging (GRAPPA accelerator factor) with a TR = 2060 ms, TE = 25 ms, slice thickness = 4 mm, with no slice gap was acquired. The resting state scan lasted 8 min 22 s (240 measurements). A high resolution T1 MPRAGE was also acquired with the following parameters; TR = 1600 ms, TE = 3.01 ms, slick thickness = 1 mm. All functional analysis was carried out using FSLv6.0.2.

### Whole brain ICA based analysis

Data were pre-processed using FEAT (fMRI expert analysis tool), a part of FSL using an automated script [26, 27]. Pre-processing includes skull stripping using brain extraction tool (BET), motion correction using MCFLIRT, slice timing correction as TR > 1 s[28], spatial smoothing (6 mm full-width half maximum) and structural registration using skull stripped image of each participant to (Montreal Neurological Institute) MNI 2 mm brain (non-linear registration) using (FMRIB linear image registration tool) FLIRT. Noise signals were identified individually and removed using ICA-AROMA toolbox [29]. ICA-AROMA incorporates probabilistic Independent Component Analysis (ICA) on the partly pre-processed single-subject fMRI data (following spatial smoothing and normalization but before high-pass filtering), identifies independent components (ICs) representing motion artifacts and removes them from the fMRI time-series using linear regression. Group-level ICA with multi-session temporal concatenation was used to delineate global functional resting-state networks. Masking of non-brain voxels, voxel-wise de-meaning of the data and normalization of the voxel-wise variance were applied to the input data. ICA maps were thresholded at 0.5. The number of components was optimized using Laplace approximation to the Bayesian evidence of the model order [30]. Results of the group-ICA correspond to statistically independent components, that is, estimates of functional resting state networks (RSNs). The set of spatial maps from the group-average analysis was used to generate subject-specific versions of the spatial maps, and associated timeseries, using “dual_regression” [31]. First, for each subject, the group-average set of spatial maps is regressed (as spatial regressors in a multiple regression) into the subject's 4D space–time dataset. This results in a set of subject-specific time series, one per group-level spatial map. Next, those time series are regressed (as temporal regressors, again in a multiple regression) into the same 4D dataset, resulting in a set of subject-specific spatial maps, one per group-level spatial map. We then tested for [group differences, etc.] using FSL's randomize permutation-testing tool using general linear model (GLM) which defined to create a multiple subject design matrix with three groups.

### ROI-based analysis

To further explore whether there is altered FC even at the level of individual brain structures, we employed time series-based analysis in the ROIs, bilateral piriform cortex (PC) and orbitofrontal cortex (OFC). The ROIs are validated and can be found in already published studies [32]. The first step includes applying appropriate transforms to the ROIs since they are defined in structural space. Hence, they cannot be used for functional analysis. This is done using fslutilites, “applywarp”. The next step is to extract the time series from the warped ROI, which is performed by “fslmeants” command. Once the time series is extracted, single subject analysis is carried out which is similar to fMRI analysis [27], followed by group level analysis for each ROI [20].

### Statistical analysis

All statistical analysis, unless stated otherwise, was carried using statistical software GraphPad Prism version 8.0. Whole brain ICA results using dual regression are by default reported at pFWE < 0.05. ROI based results are reported using Z statistic images which were thresholded non-parametrically using clusters determined by Z > 3.1 and a (corrected) cluster significance threshold of p < 0.05 [33].

## Results

### Age, gender, olfactory sensitivity

A one-way ANOVA showed no age-based differences between groups *(F (2, 141)* = *0.13, p* = *0.88)*. In addition, post-hoc multiple comparisons (Tukey) revealed no significant differences among groups. A non-significant chi-square test showed no differences in gender distribution among the groups *[X*^*2*^* (1.75, 2* = *0.41)]*. Also, no significant differences were found for olfactory threshold scores among the groups using ANOVA with multiple comparisons *[F (2, 141)* = *1.16, p* = *0.31]*. The demographics can be seen in Table 1.

### Whole brain ICA based results

**Fig. 2:**
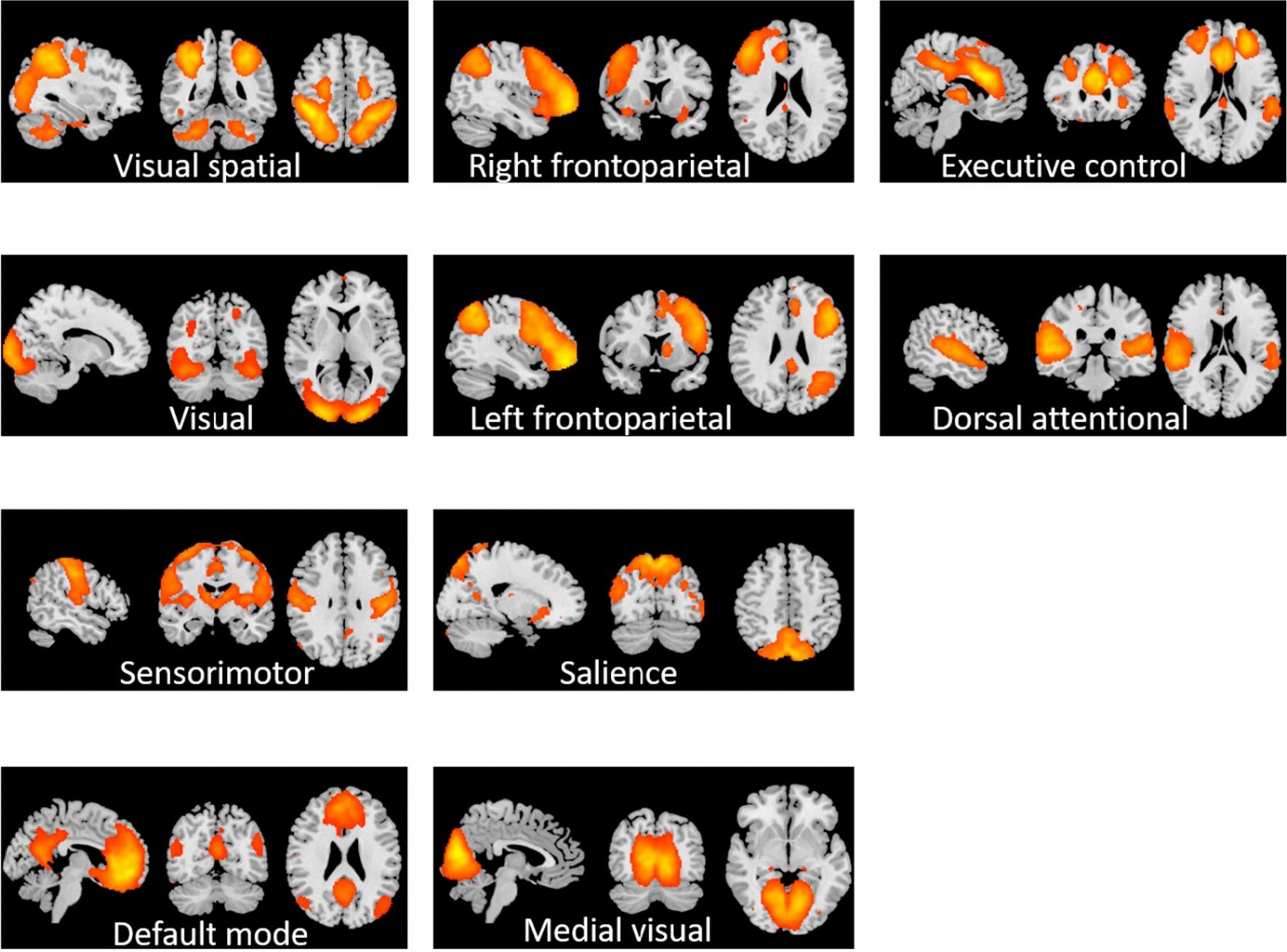
Group ICA results depicting 10 RSNs identified. Using Group Independent component analysis, 10 resting state networks were identified

**Fig. 3:**
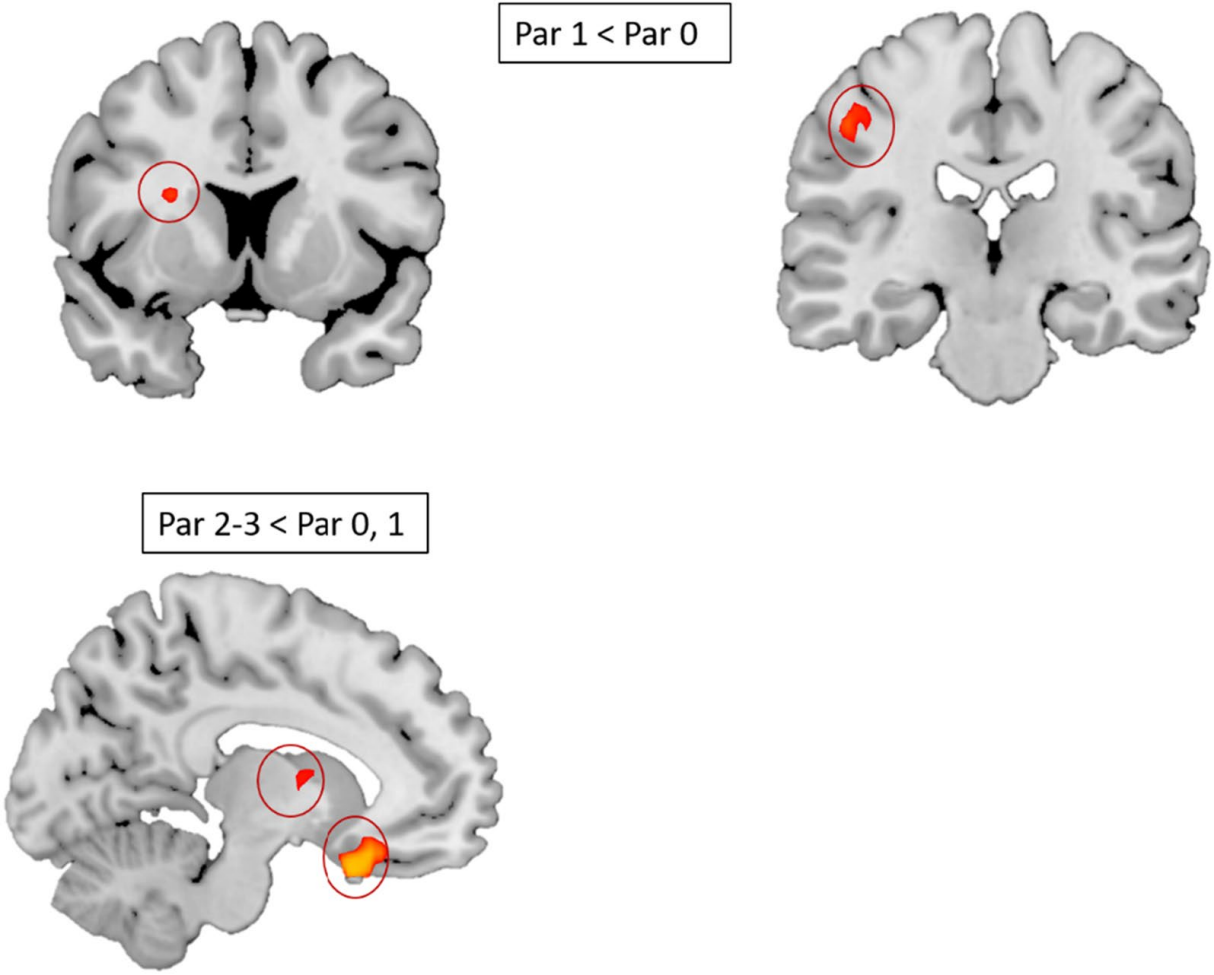
Group level results post dual regression. For the contrast par 1 < par 0., all in red circle, part of caudate nucleus, putamen and shows supramarginal gyrus having lower coherence in Par 1. Par 2–3 < Par 0 and also for Par 2–3 < Par 1, shows thalamus and subcallosal cortex having lower coherence in Par 2–3

A total of ten components (Fig. 2) were identified as RSNs from a group ICA analysis which included, visual network, default mode network (DMN), executive control network, sensorimotor network, medial visual network, left and right fronto-parietal network, visuo-spatial network, dorsal attentional network [34] and salience network [35]. The regions in the Salience network and Executive control network, in which Par 1 group showed significantly lower temporal concatenation (coherence) as compared to Par 0 group, include regions surrounding caudate nucleus and putamen and supramarginal gyrus (Fig. 3, top). Similarly, regions in the Medial visual network, in which Par 2–3 group showed significantly lower temporal concatenation as compared to both Par 0 and Par 1 group include bilateral thalamus and subcallosal cortex (Fig. 3, bottom). For other contrasts, brain activations did not survive multiple comparisons.

### ROI based results

**Fig. 4:**
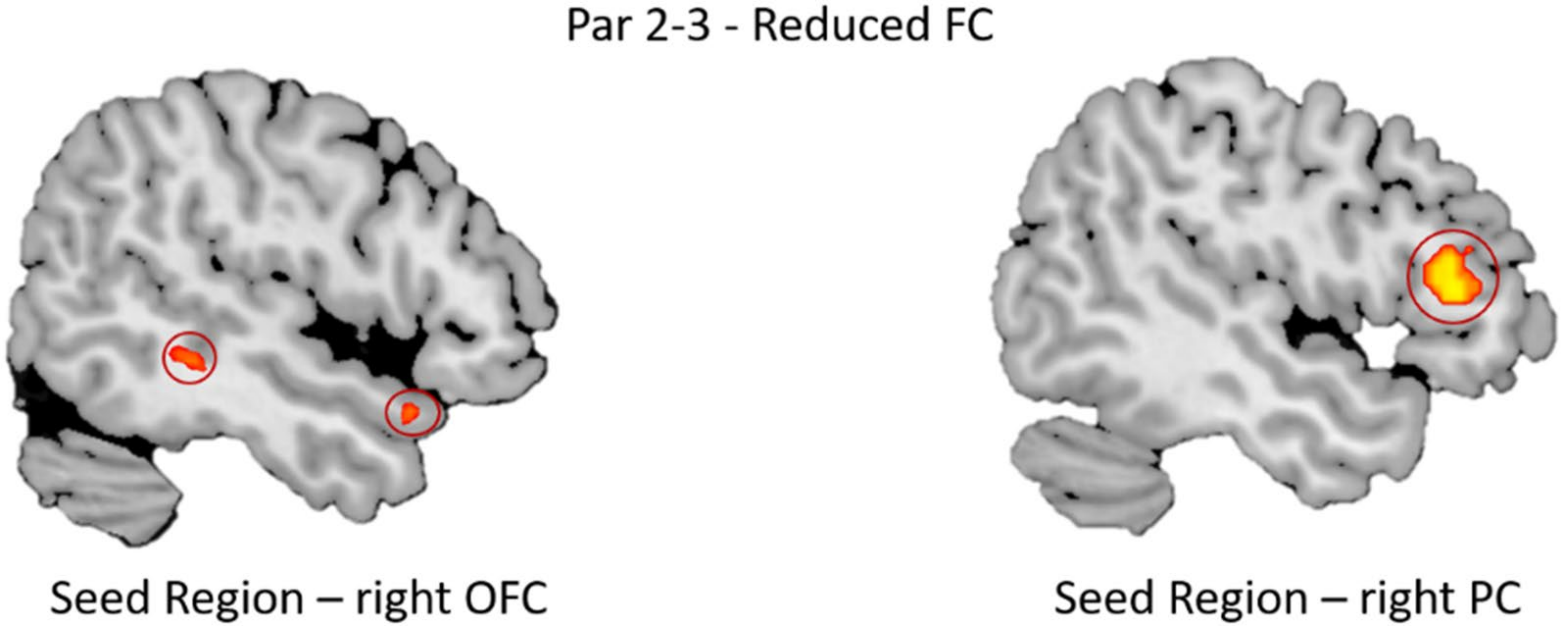
Shows results from ROI based approach. In Par 2–3, Seed region right orbitofrontal cortex (OFC) had reduced functional connectivity with areas of middle temporal gyrus and temporal pole and for, seed region right piriform cortex (PC), dorso-lateral pre-frontal cortex had reduced functional connectivity (FC)

Piriform cortex (PC) and orbitofrontal cortex (OFC) were the regions chosen to carry out ROI-based FC chosen to understand the effect of parosmia on primary and secondary olfactory areas. Comparing groups, for seed right OFC: temporal pole and supramarginal gyrus were found to be significant and more functionally connected in Par 1 as compared to Par 2–3 (Fig. 4). Similarly, for the right PC, dorso-lateral pre-frontal cortex (dlPFC) was significantly more connected in Par 0 as compared to Par 2–3 (Fig. 4). For other contrasts, brain activations did not survive multiple comparisons.

## Discussion

Using resting state fMRI, patients with a higher degree of parosmia were found to have reduced FC in both whole-brain as well as in ROI-based analyses. Patients with a higher degree of parosmia (Par 2–3) showed reduced FC in medial visual network with thalamus and subcallosal cortex whereas Par 1 patients compared to Par 0 showed reduced FC in salience networks as well as executive control networks with areas surrounding putamen, caudate nucleus and supramarginal gyrus.

Group ICA resting-state fMRI studies have consistently found default mode network (DMN) which is widely regarded as rest network and its identification also serves to ascertain whether a resting state fMRI was performed [36]. Presence of DMN in our study confirms that (Fig. 1). When comparing patients with parosmia using dual regression, Salience network (SN) and Executive control were found to be significantly affected. Salience Network is defined as a group of brain areas that identify the most homeostatically relevant stimuli from a range of stimuli presented to the central nervous system. In contrast, Executive control network (ECN) has been described as brain regions that work on the chosen salience stimuli and direct attention to the stimuli while behavioral choices are being made[37]. A decrease in FC, when comparing Par 0 vs Par 1,was observed in parts of the caudate nucleus and putamen (part of ECN) and supramarginal gyrus (part of SN), which is consistent with previous studies on depression and social anxiety disorders [38, 39]. We did not quantify depression and cognitive disorders in the patients. However, because patients with parosmia often show signs of depression the observed changes in FC may be interpreted as a reflection of depressive symptoms.

When comparing Par 1 and Par 2–3, reduced FC was observed in thalamus and subcallosal cortex with Medial visual network (MVN). The subcallosal cortex has been linked to processing of emotional information in humans when viewing faces [40]. Importantly, the left subcallosal cortex was seen in emotions more related to disgust and the right subcallosal cortex in happiness. Reduced FC in the subcallosal cortex in patients with a higher degree of parosmia (Par 2–3) may suggest the feeling of disgust caused as a result of frequent parosmic episodes. Reduced FC has been seen in other modalities, like vision and audition [41], however, in MVN, the role in olfaction is understudied. The thalamus comprises of numerous nuclei with each nucleus having a distinct functional role [42]. Decreased FC was observed between the area surrounding the anterior as well as the medial dorsal part of thalamus and MVN in patients with a higher degree of parosmia (Par 2–3). A recent study contemplated the role of visual network and olfaction and found that olfaction modulates low-level visual inputs [43]. The authors of the paper focus on the cross-modal effect of various senses. In the current context it would mean strong visual memory recall without inhibition affecting patients with parosmia. The anterior part of the thalamus plays a critical role in learning and shares functions with the hippocampus [44] whereas the medial dorsal part of the thalamus is involved in olfactory processing including odor perception, discrimination, learning and attention [45]. Reduced FC in the thalamus could suggest the absence of feedback to the OFC leading to high intensity disgust perception in parosmia. It has been suggested that although there is no direct input from olfactory sensory neurons to the thalamus, it would be directly involved in the processing of odors by having a reverse connection with many olfactory regions such as the amygdala (e.g., emotional valence) and OFC (e.g., odor intensity) [46]. The fact that patients with a higher degree of parosmia have reduced FC may suggest two things: first, reduced subcallosal activity may relate to the enhanced perception of disgust and second, reduced thalamic input may lead to increased intensity of the percept due to inhibited feedback.

Region of interest (ROI) analysis was performed to look into the functional connectivity (FC) and comparing differences in activations with seed region among the groups. The ROI analysis provides a possibility of measuring functional connectivity between brain regions [47–49]. We used the bilateral piriform cortex (PC) and OFC as seed regions for the analysis because both regions are regarded as significant primary and secondary olfactory areas, respectively [50]. The ROI analysis was initiated because the distorted smell perception in patients with parosmia likely involves primary and secondary olfactory cortices. FC was affected in Par 2–3 for both right PC and OFC, where reduced FC was seen between right PC and dlPFC and between right OFC and temporal pole and supramarginal gyrus. It has been shown that dlPFC is involved in memory encoding [51], response representation towards tasks [52] and occasionally related to decision making about the intensity of stimuli [53]. dlPFC has also been implicated in body odor encoding task along with OFC [54]. Reduced FC between right PC (primary olfactory area) and dlPFC (decision making center) could explain increased “disgust” or “distorted” feeling associated with parosmia in Par 2–3.

The right OFC, on the other hand, had reduced FC between the temporal pole and the supramarginal gyrus in Par 2–3. Both areas are important in olfactory processing. The temporal pole is often regarded as a paralimbic region involved with semantic processing and socio-emotional processing which via uncinate fasciculus provides a direct bidirectional path to the orbitofrontal cortex, allowing mnemonic representations stored in the temporal pole to bias decision making in the frontal lobe [55]. A recent study using diffusion neuroimaging has found direct connections between OFC and the temporal pole, which is involved in visual, auditory and semantic functions [56]. A reduced FC between the temporal pole and the OFC could imply, as parosmia worsens, that decision making related to the emotional load of an odor mediated by the OFC becomes less clear, leading to an increase/amplification of the distortions. The supramarginal gyrus also has functional associations with the OFC. It is regarded as a crucial part of the somatosensory network. Reduced FC between these two structures in Par 2–3 could result in negative emotional effects as seen in previous study related to masked vs non-masked body odors [57]. The authors of this study found supramarginal activations in response to a negative outcome associated with a real-life incongruent moral dilemma. This could explain “distorted” smell perception in parosmia which may be caused or amplified by a reduction in functional information transfer between different brain regions.

### Limitations

Absence of an objective and standardized assessment tool for parosmia poses a significant challenge. As one has to rely on self-reported altered olfactory perceptions, it introduces subjectivity and lack of uniformity in evaluation. Another limitation of the study is the failure to include a depression measuring questionnaire for factoring in affects leading to changes in resting state FC.

## Conclusions

A higher degree of parosmia is linked with reduced functional connectivity seen at the level of both, whole brain as well as a ROI based approach. At the level of the whole brain, observed changes in FC are compatible with enhanced perception of disgust. These perceptions are even seen at the level of individual brain regions. Overall, the present results suggest that higher degrees of parosmia are associated with, enhanced perception of “disgust” and distortion of odor guided decision-making possibly due to changes in information transfer between key relays of the olfactory system.

## Data Availability

The data underlying this article were provided by DY and AA under licence / by permission. Data will be shared on request to the corresponding author with permission of DY and AA.

## Abbreviations

FC: Functional connectivity
OD: Olfactory dysfunction
BOLD: Blood oxygenated level dependent
OFC: Orbitofrontal cortex
PC: Piriform cortex
